# Transcriptional Reprogramming of Pea Leaves at Early Reproductive Stages

**DOI:** 10.3389/fpls.2019.01014

**Published:** 2019-08-07

**Authors:** Karine Gallardo, Alicia Besson, Anthony Klein, Christine Le Signor, Grégoire Aubert, Charlotte Henriet, Morgane Térézol, Stéphanie Pateyron, Myriam Sanchez, Jacques Trouverie, Jean-Christophe Avice, Annabelle Larmure, Christophe Salon, Sandrine Balzergue, Judith Burstin

**Affiliations:** ^1^Agroécologie, AgroSup Dijon, Institut National de la Recherche Agronomique, Université Bourgogne Franche-Comté, Dijon, France; ^2^IPS2, Institute of Plant Sciences Paris-Saclay (Institut National de la Recherche Agronomique, Centre National de la Recherche Scientifique, Université Paris-Sud, Université d'Evry, Université Paris-Diderot, Sorbonne Paris-Cité, Université Paris-Saclay), POPS-Transcriptomic Platform, Saclay Plant Sciences (SPS), Orsay, France; ^3^Normandie Université, Institut National de la Recherche Agronomique, Université de Caen Normandie, UMR INRA–UCBN 950 Ecophysiologie Végétale et Agronomie, SFR Normandie Végétal FED 4277, Caen, France

**Keywords:** legumes, leaves, reproductive period, nitrogen remobilization, transcriptomics, co-expression, transcription factors, transporters

## Abstract

Pea (*Pisum sativum* L.) is an important source of dietary proteins. Nutrient recycling from leaves contributes to the accumulation of seed proteins and is a pivotal determinant of protein yields in this grain legume. The aim of this study was to unveil the transcriptional regulations occurring in pea leaves before the sharp decrease in chlorophyll breakdown. As a prelude to this study, a time-series analysis of ^15^N translocation at the whole plant level was performed, which indicated that nitrogen recycling among organs was highly dynamic during this period and varied depending on nitrate availability. Leaves collected on vegetative and reproductive nodes were further analyzed by transcriptomics. The data revealed extensive transcriptome changes in leaves of reproductive nodes during early seed development (from flowering to 14 days after flowering), including an up-regulation of genes encoding transporters, and particularly of sulfate that might sustain sulfur metabolism in leaves of the reproductive part. This developmental period was also characterized by a down-regulation of cell wall-associated genes in leaves of both reproductive and vegetative nodes, reflecting a shift in cell wall structure. Later on, 27 days after flowering, genes potentially switching the metabolism of leaves toward senescence were pinpointed, some of which are related to ribosomal RNA processing, autophagy, or transport systems. Transcription factors differentially regulated in leaves between stages were identified and a gene co-expression network pointed out some of them as potential regulators of the above-mentioned biological processes. The same approach was conducted in *Medicago truncatula* to identify shared regulations with this wild legume species. Altogether the results give a global view of transcriptional events in leaves of legumes at early reproductive stages and provide a valuable resource of candidate genes that could be targeted by reverse genetics to improve nutrient remobilization and/or delay catabolic processes leading to senescence.

## Introduction

Grain legumes accumulate large amounts of proteins in their seeds, which are widely used for human and animal nutrition. In legumes, symbiotic nitrogen fixation, and nitrate uptake by roots are two complementary modes of nitrogen acquisition that decline during the reproductive period (Salon et al., 2001). Nitrogen stored in plant parts is then remobilized to sustain seed protein accumulation. The contribution of nitrogen remobilization to seed protein yield varies from 45 to 90%, depending on the species and conditions (Warembourg and Fernandez, 1985; Kurdali et al., 1997). In pea (*Pisum sativum* L.), 70% of the amount of nitrogen in mature seeds is derived from remobilization processes (Jensen, 1987; Schiltz et al., 2005). The chloroplast enzyme ribulose-1,5-bisphosphate carboxylase/oxygenase, which plays an essential role in carbon fixation, is one major source of nitrogen in leaves (Jiang et al., 1993). Its degradation starts before leaf senescence, a catabolic process leading to yellowing, chloroplast disassembly, and finally cell death (Kohzuma et al., 2017). Because most leaf nitrogen is stored in the form of proteins with roles in the photosynthetic machinery, nitrogen remobilization may affect photosynthetic activities, which may curtail the reproductive period and limit seed yield. Nutrient deficiencies, high temperature and drought, are environmental factors accelerating leaf senescence, thereby shortening the reproductive period and impacting negatively seed filling (Olsson, 1995; Srivalli and Khanna-Chopra, 1998). Stay-green varieties, where leaf senescence is delayed, are used in some cereal improvement programs since they display a greater grain yield under post-anthesis drought (Borrell et al., 2001). However, stay-green phenotypes are not necessarily associated with higher yields, especially when chlorophyll catabolism is blocked since the active degradation of chlorophyll is a prerequisite for nitrogen remobilization from the pigment-associated proteins (Thomas, 1997; Thomas and Howarth, 2000). Hence, optimizing the balance between nutrient recycling and leaf longevity is necessary to increase and stabilize protein yield. This requires the identification of the underlying molecular determinants that could be targeted in breeding programs for higher and stable protein yields.

The mechanisms controlling nutrient recycling have been mainly studied during senescence associated with leaf yellowing. Genes up-regulated during this process, generally referred to as senescence-associated genes (SAGs) or senescence-enhanced genes, were identified (Buchanan-Wollaston et al., 2005). Several SAGs are related to autophagy, a vesicular trafficking process that regulates nutrient recycling and remobilization by participating in the methodical degradation of the cell constituents (Masclaux-Daubresse et al., 2017). Several lines of evidence indicate that senescence-related transcription factors (TFs) can directly regulate autophagy genes in plants (Garapati et al., 2015). Transcriptomics revealed that a large number of NAC (no apical meristem, transcription activation factors, and cup-shaped cotyledon) TFs are expressed during leaf senescence (Balazadeh et al., 2010; Breeze et al., 2011; Yang et al., 2016). Functional studies in Arabidopsis showed that NACs can act as positive or negative regulators of senescence (Yang et al., 2011; Liang et al., 2014; Garapati et al., 2015; Zhao et al., 2015; Pimenta et al., 2016). However, we are far from a comprehensive understanding of the pathways and regulatory networks influencing nutrient recycling in crops, especially in grain legumes such as pea, a monocarpic species that exhibits different patterns of whole plant senescence compared to Arabidopsis, and in which the production of seeds triggers nutrient remobilization (Noodén and Penney, 2001; Pic et al., 2002). The aim of the present study was to unveil the transcriptional reprogramming of pea leaves at stages preceding the sharp decrease in chlorophyll breakdown. Nitrogen remobilization between tissues was highly dynamic during this period, as shown through a time-series analysis of the translocation of ^15^N absorbed in the form of nitrate up to flowering. Leaves of the vegetative and reproductive nodes were analyzed by transcriptomics and a gene co-expression approach was used to highlight potential regulators of specific biological processes. The same approach in the fodder legume species *M. truncatula* revealed a number of shared co-expression modules.

## Materials and Methods

### Plant Growth Conditions

Pea (*Pisum sativum* L, genotype “Caméor”) and *Medicago truncatula* (*M. truncatula*, Gaertn., A17 genotype) plants were grown in a greenhouse under controlled temperature (at least 18°C during the day and 15°C during the night) and photoperiod (16h/d). *M. truncatula* seeds were scarified and vernalized 4d at 5°C before sowing. Plants were grown in 7L (pea) or 3L (*M. truncatula*) pots containing 40% attapulgite and 60% clay balls. Plants were not inoculated with Rhizobia. Nitrogen nutrition of all plants relied on the absorption of nitrate for the purpose of long-term ^15^N-labeling. Two nitrogen availability conditions were used. Control plants (N+) were supplied with the nutrient solution previously described (Zuber et al., 2013) until tissue collection. For N– plants, nitrate was depleted at the beginning of flowering using the same solution without KNO_3_ and Ca(NO_3_)_2_ (replaced by 1.85 mM KCl and 0.25 mM CaCl_2_). Leaf chlorophyll content at the first flowering node was measured using a SPAD-502 chlorophyll meter on 12–16 plants per condition and stage (Minolta Camera Co. Ltd., Japan). The plant, pod and seed characteristics in Table S1 were measured at maturity (63 days after flowering) from eight biological replicates (i.e., individual plants). An analysis of variance was performed to reveal significant effects of nitrogen limitation on these traits (Statistica v7.0 software).

### Dynamic of Nitrogen Remobilization at the Whole Plant Level in Pea and *M. truncatula*

For each time point [beginning of flowering, 14, 27, and 63 days after flowering (DAF)], six plants were used per condition (N+, N–): four plants were supplied with the nutrient solutions described above labeled with 3 atom% excess of ^15^N (as K^15^NO_3_) until flowering (i.e., 35 days labeling), and two unlabeled plants served to estimate natural ^15^N abundance. The pots were organized in a randomized complete-block design. For each time point and condition, leaves of the vegetative nodes (lower leaves), and reproductive nodes (upper leaves), stems, roots, pods (*M. truncatula*), seeds, and pod wall (pea) were harvested separately. The dry matter of each tissue was determined after oven-drying at 80°C for 48 h. All tissues were ground using the cutting mill SM200 (Retsch, Haan, Germany), then using the ZM 200 grinder (Retsch). Total N and ^15^N/^14^N ratio were determined from 5 mg powder using a PDZ Europa ANCA-GSL elemental analyzer interfaced to a PDZ Europa 20-20 isotope ratio mass spectrometer (Sercon Ltd., Cheshire, UK). The calculation of endogenous nitrogen (i.e., stored during the vegetative phase) remobilized across plant tissues between two developmental stages was determined from elemental and isotope amounts in the different organs using the PEF (Plant Elemental Flux) tool developed in visual basic applications (Salon et al., 2014). The quantitative values for nitrogen remobilized (mg) from or to each tissue between two time points were subjected to a *t*-test using Statistica software (v7.0) to reveal significant effects of nitrogen deficiency on the quantity of nitrogen remobilized from each tissue.

### Leaf Samples and RNA Extraction

Lower and upper leaves were collected from 6 to 8 individual plants deprived or not of nitrate, at three stages: flowering, 14 and 27 DAF. The absence of nodules on the root system was checked at the time of tissue collection. Lower leaves corresponded to leaves of the two last vegetative nodes and upper leaves corresponded to leaves of nodes carrying flowers at the flowering stage, and to leaves of the third and fourth reproductive nodes at 14 and 27 DAF. The leaf samples were immediately frozen in liquid nitrogen, then stored at −80°C. RNA was extracted from 100 mg of frozen powder using the RNeasy Plant Mini Kit according to manufacturer's protocol (Qiagen, Courtaboeuf, France). RNA quality was checked on agarose gel 1.5%, then using the Agilent 2100 Bioanalyzer.

### RT-qPCR Using *ELSA* as Indicator of Leaf Senescence

For profiling the expression of the *Early Leaf Senescence Abundant cysteine protease gene (ELSA)* (Pic et al., 2002) by RT-qPCR, leaf samples collected at flowering, 14 and 27 DAF on plants deprived or not of nitrate (*n* = 6–8) were used. RT-qPCR was performed with the iScript cDNA synthesis kit according to manufacturer's protocol (Bio-Rad, Marnes-la-Coquette, France) and the GoTaq qPCR Master Mix (Promega, Charbonnières, France) using 10 ng cDNA and 0.2 μM of each primer in a final volume of 5 μl. Analyzes were performed in triplicates from each biological replicate using the LightCycler 480 system (software v1.5.0, Roche, Meylan, France) as previously described (Zuber et al., 2013). The normalization method was ΔΔct using actine, histone, and EF1 α as reference genes (primers in Table S2). Analyses of variance and Student-Newman-Keuls (SNK) tests using the Statistica software (v7.0) revealed significant changes in gene expression between stages and/or in response to nitrate deficiency.

### Transcriptomics of Leaves and Validation by RT-qPCR

Three biological replicates of leaves from vegetative and reproductive nodes were subjected to transcriptomics. Pea NimbleGen-microarrays were developed to profile expression of 40795 sequences: 40454 mRNA originating from the PsCameor_Uni_Lowcopy set (Alves-Carvalho et al., 2015), 323 putative precursors of miRNA predicted in the “Test assembly multiple k-mer” contig set (Alves-Carvalho et al., 2015), and 18 controls. Two specific oligonucleotides were used for each mRNA sequence and one oligonucleotide was used per miRNA precursor sequence (forward and reverse). These probes were spotted in triplicates on the GENOPEA array. *M. truncatula* NimbleGen-microarrays (Herrbach et al., 2017) were used in parallel. They represent 83029 probes (spotted in triplicates) corresponding to transcribed regions of the *M. truncatula* genome from the Symbimics program (https://iant.toulouse.inra.fr/symbimics/). The Ambion MessageAmp™ II aRNA Amplification Kit was used to amplify sufficient amounts of copy RNA extracted, as described above, from upper leaves and lower leaves of three biological replicates (independent plants). The Double stranded cDNA synthesis was realized using T7-oligo-dT and the antisense RNA (aRNA) was created by *in vitro* transcription according to manufacturer's protocol (Life technologies SAS, Saint Aubin, France). The labeling with Cy3 or Cy5 was performed by reverse transcription of aRNA using labeled nucleotides (Cy3-dUTP or Cy5-dUTP, Perkin-Elmer-NEN Life Science Products). For each nutritional condition and leaf type, the following co-hybridizations were performed: 14 DAF vs. flowering, 27 DAF vs. 14 DAF. For each comparison, a dye swap was realized. The hybridization of labeled samples on the slides, scanning and data normalization were performed as previously described (Lurin et al., 2004). Differential analysis was based on the log_2_ ratios averaged on the dye-swap: the technical replicates were averaged to get one log_2_ ratio per biological replicate and these values were used to perform a paired *t*-test. The raw *P*-values were adjusted by the Bonferroni method, which controls the family wise error rate, and probes were considered as differentially expressed when the Bonferroni corrected *P*-value was <0.05. Transcriptome datasets were deposited in the NCBI Gene Expression Omnibus database with the accession numbers GSE109789 for pea and GSE109521 for *M. truncatula*. All pea sequences with “PsCam” accession numbers could be retrieved from the pea RNAseq gene atlas at http://bios.dijon.inra.fr/ (PsUniLowCopy data set).

Twenty genes differentially regulated between two stages were selected for RT-qPCR analyses (as describe above) in leaves from three biological replicates of plants well-supplied with nitrate. For each leaf sample (lower and upper leaves) and developmental period (14 DAF vs. flowering, 27 vs. 14 DAF), Pearson's correlation coefficient (r) between microarray and RT-qPCR expression levels were calculated (Table S3, primers in Table S2). Hierarchical clustering of transporter and TF genes was performed using the Genesis software (v1.8.1; default parameters) (Sturn et al., 2002). Gene Ontology (GO) term enrichment analysis was performed using topGO (elim method and Fisher's exact test) in Bioconductor v2.9 implemented in BIOS (Architecture BioInformatique Orientee Services, http://bios.toulouse.inra.fr/). Phylogenetic trees were generated from protein sequences using the Neighbor-joining method of the ClustalW2 program available at https://www.ebi.ac.uk/Tools/phylogeny/. Orthologous genes between pea and *M. truncatula* (v4.02) were identified using OrthoFinder v1.1.8 (MCL clustering algorithm and DIAMOND v0.9.10.111 for the alignment with default parameters). Of the 19055 clusters identified, 15445 were retained for transcriptome comparisons because they were made of a unique gene per species (14980 sequences with probes on the arrays).

### Gene Co-expression Network Construction

Log_2_ intensity values from each red and green channels were normalized based upon quantiles using the preprocess Core package (v1.34.0) available in R (v3.3.1). Gene variance was calculated using the gene filter R package (Gentleman et al., 2018) (v1.54.2) and only sequences displaying a variance >0.2 were retained for co-expression studies. Gene co-expression networks were built using the Expression Correlation plugin (v1.1.0, http://apps.cytoscape.org/apps/expressioncorrelation) of Cytoscape (v3.5.1) (Cline et al., 2007). We have chosen r cut-off of 0.95 and −0.95 (*r*^2^ >0.9) to build P-REMONET from the pea transcriptome dataset, and of 0.90 and −0.90 (*r*^2^ >0.81) to build M-REMONET from the *M. truncatula* transcriptome dataset. The node degree of the networks followed a power-law distribution. A Prefuse Force Directed layout was used to visualize the entire networks in Cytoscape. For ease of visualization of TF-related modules, the genes connected to the TFs were organized using the Circular Layout algorithm.

## Results

### Dynamics of Nitrogen Remobilization During the Reproductive Phase in Pea

**Figure 1:**
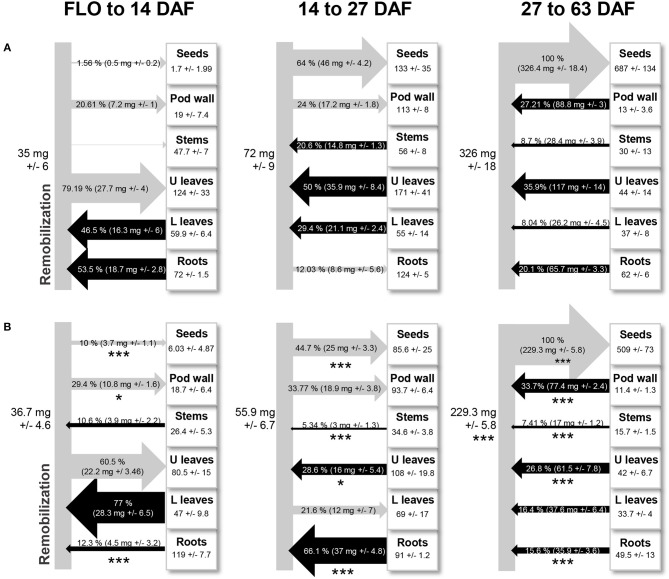
Nitrogen remobilized between plant parts during the reproductive phase in pea, with **(A)** or without **(B)** nitrate supply from flowering. Black arrows indicate that nitrogen is remobilized from a tissue, and gray arrows indicate that nitrogen is redistributed toward a tissue. In squares are mean values of nitrogen quantity in each compartment (roots, leaves of the vegetative nodes = L leaves, leaves of the reproductive nodes = U leaves, stems, pod wall, and seeds) at the last stage (14, 27, and 63 days after flowering). Data are mean values ± standard errors. In each diagram and for each tissue, stars indicate significant variations in the amount of nitrogen remobilized in response to nitrogen deprivation: *P < 0.1, **P < 0.05, ***P < 0.01 (*t*-test, data from 4 individual plants).

An overview of nitrogen remobilization between tissues was obtained through a time-series analysis of the translocation of ^15^N absorbed in the form of nitrate during the vegetative phase (Figure 1). From the beginning of flowering to seed filling in the first pods (14 days after flowering, DAF), nitrogen taken up during the vegetative period was remobilized from leaves below the first flowering node (lower leaves; 46.5%, Figure 1A) and roots (53.5% of the total amount of remobilized nitrogen). This pool of nitrogen was mainly redistributed toward leaves of the reproductive part (upper leaves) and to pod walls. Then, from 14 DAF until the end of 1st pod seed filling (27 DAF), nitrogen was remobilized from stems (20%), lower, and upper leaves (80%) to seeds, pod wall, and roots. Roots behave as a transient sink of nitrogen during this period, probably because leaves, and stems provide sufficient amounts of nitrogen to fulfill seed nitrogen requirements. At later stages (27–63 DAF), nitrogen was remobilized from all tissues to seeds, which at maturity contained 54% of nitrogen derived from remobilization processes (Figure 1A). This shift to systemic remobilization to seeds coincided with the beginning of chlorophyll degradation in leaves (starting 33 DAF, Figure S1A). The increased expression of the early senescence marker *ELSA* in lower and upper leaves 27 DAF was indicative of a molecular switch toward proteolysis (Figure S1B). The 4-fold higher expression of *ELSA* in upper leaves 27 DAF, compared to lower leaves, suggests higher proteolytic activities in these leaves. Altogether, the data indicate that 27 DAF is a transition stage toward leaf senescence.

Nitrate deficiency during the reproductive phase triggered major changes in the dynamics of nitrogen remobilization (Figure 1B). From flowering to 14 DAF, nitrogen remobilization from roots decreased while nitrogen remobilization from lower leaves increased significantly in response to nitrate deficiency. From 14 to 27 DAF, roots became the major source of nitrogen specifically under nitrate deficiency and nitrogen remobilization from other tissues was significantly reduced in that condition, especially from lower leaves that became a transient sink for nitrogen. This may be part of the mechanisms used by plants to avoid precocious senescence in response to nitrogen deficiency. While leaf nitrogen content decreased continuously from flowering to maturity under nitrate-sufficient conditions, it remained unchanged between 14 and 27 DAF in nitrate-deprived plants (Figure S2). These data and the lower expression of *PsELSA* in lower and upper leaves of these plants, suggest a lower remobilization rate in response to nitrate deficiency (Figure S1B), associated with a maintained chlorophyll content (Figure S1A). At later stages (27–63 DAF), nitrogen remobilization from almost all tissues was significantly reduced in response to nitrate deficiency and, at maturity, these plants were characterized by a reduced seed yield and one-seed weight (Table S1).

### Transcriptome Changes in Pea Leaves at Early Reproductive Stages

**Figure 2:**
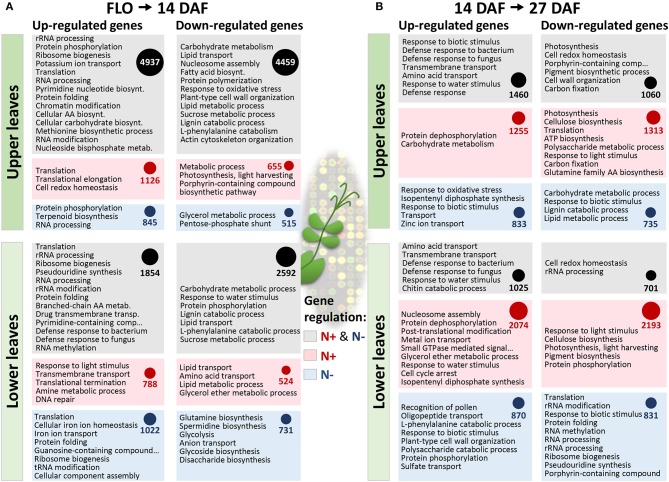
Transcriptome changes in leaves of vegetative (lower leaves) and reproductive (upper leaves) nodes during pea seed development and in response to nitrate availability. The number of genes whose expression varied (Bonferroni-corrected *P*-value < 0.05) between the beginning of flowering and 14 days after flowering (**A**, 14 DAF) and/or between 14 and 27 days after flowering (**B**, 27 DAF). The number of genes whose expression varied regardless of nitrate nutrition are shown in gray boxes, while the number of genes whose expression varied specifically under nitrate-supply (N+) or nitrate-deprivation (N–) are shown in red and blue boxes, respectively. The circles are proportional to the number of genes in each box. GO terms significantly enriched (Fisher *P* values < 0.005) in each gene list are sorted according to *P* values (lowest at the top).

**Figure 3:**
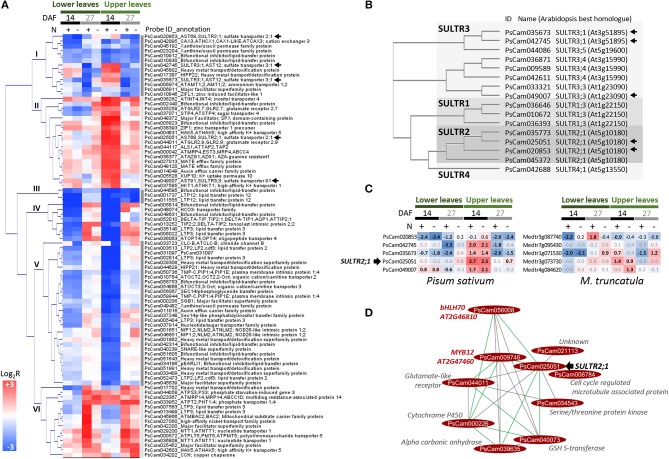
Transporter genes differentially expressed (≥4-fold) in pea leaves between at least two stages. **(A)** Hierarchical clustering of expression profiles in lower and upper leaves. The color scale indicates Log_2_R for the comparisons 14 DAF vs. flowering (14), 27 DAF vs. 14 DAF (27), and in response to nitrate-deficiency (N–). Arrows indicate sulfate transporter (SULTR) genes. **(B)** Phylogenetic tree of SULTR sequences identified in the Pea RNAseq Atlas (Alves-Carvalho et al., 2015). **(C)** Expression (array data) of pea SULTR genes marked by an arrow in **(A,B)** and expression profile of the *Medicago truncatula (M. truncatula)* orthologs (In bold, Bonferroni-corrected *P*-value < 0.05). **(D)** Genes connected to PsCam025051/SULTR2;1 in P-REMONET.

The molecular processes regulated in pea leaves at stages characterized by dynamic nitrogen remobilization between tissues, from flowering to 27 DAF, were investigated by transcriptomics. An analysis of transcriptome changes occurring in leaves of the vegetative and reproductive nodes under both nitrate-sufficient and -deficient conditions was carried out. The GENOPEA array representing 40777 pea sequences was used. Quantitative RT-PCR data for 20 genes differentially expressed showed high correlations with array data (Pearson's correlation coefficient r ranging from 0.80 to 0.93, Table S3), confirming the robustness of the approach to identify genes differentially regulated in pea leaves. An analysis of gene ontology (GO) terms significantly enriched (Fisher's *P*-value<0.005) in the lists of genes differentially regulated during the time course provided an overview of the biological processes activated or repressed (Figure 2). Major changes occurred in the upper leaf transcriptome from flowering to 14 DAF regardless of nitrate supply. Between 14 and 27 DAF, 2074 and 2193 genes were, respectively, up- and down-regulated in lower leaves specifically under nitrate supply. Many GO terms in Figure 2 are related to transport processes. Expression patterns and annotations of the 678 transport-related probes differentially regulated between at least two developmental stages are presented in Table S4, thus providing a set of candidate genes for controlling the transfer of nutrients. The most differentially regulated genes (more than 4-fold) are presented in Figure 3A. These 88 genes were classified into six main clusters based on hierarchical clustering of their expression patterns. GO analysis revealed an over-representation of genes encoding transporters of sulfate (SULTR), metal ions, and lipids. The previously reported role of sulfate-derived molecules in controlling autophagy and SAGs (Álvarez et al., 2012; Yarmolinsky et al., 2014) prompted us to study the expression and homologies of *SULTR* genes. A phylogenetic tree based on alignments of all SULTRs present in the Pea Gene Atlas (Alves-Carvalho et al., 2015) and a search for the well-characterized Arabidopsis homologs revealed that the differentially regulated genes belong to groups 2 and 3 of low-affinity SULTR (Figure 3B). Of the five differentially regulated SULTR genes, four were up-regulated in leaves of the reproductive nodes 14 DAF (Figure 3C), suggesting they could contribute to sulfate transport in these leaves.

### TF Genes Differentially Regulated in Pea Leaves Between Stages

**Figure 4:**
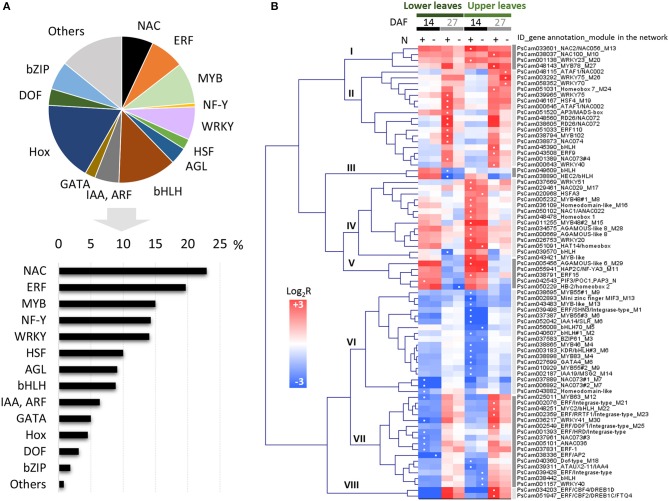
Genes with GO terms related to TF activity (GO:0003700) and regulation of transcription (GO:0045449) differentially expressed at least 4-fold in pea leaves between two developmental stages. **(A)** Number of differentially expressed genes represented as a percentage (bars) of the total number of genes per TF family (pie chart). **(B)** Hierarchical clustering of their expression profiles in lower and upper leaves. The color scale indicates Log_2_R for the comparisons 14 DAF vs. flowering (14), 27 DAF vs. 14 DAF (27), and in response to nitrate-deficiency (N–), and white dots in squares indicate the highest values. MYB, myeloblastosis; NF-Y, nuclear factor Y; HSF, heat shock transcription factor; AGL, agamous-like; bHLH, basic helix-loop-helix; IAA, indoleacetic acid-induced protein; ARF, auxin response factor; Hox, Homeodomain/homeobox; DOF, DNA-binding One Zinc Finger; bZIP, Basic Leucine Zipper.

To identify putative regulators in pea leaves, genes belonging to the categories “TF activity” (GO:0003700) and “regulation of transcription” (GO:0045449), and significantly regulated between at least two stages, were selected. The annotation and expression patterns of these 625 probes are available in Table S5. We subsequently focused on the 78 TF genes displaying more than a 4-fold change in expression. They belonged to various families, the most enriched TF families in this dataset being NAC and ethylene response factor (ERF), followed by myeloblastosis (MYB), nuclear factor Y (NF-Y), and WRKY TFs (Figure 4A). These were classified into eight main clusters based on hierarchical clustering of their expression patterns (Figure 4B). The regulation of *NAC* and *ERF* genes suggested specialized functions at early or late stages and/or in leaves at specific positions. For example, while *NAC2/PsCam033601* and *NAC100/PsCam038037* were up-regulated in all sample comparisons, *NAC1/PsCam050102* expression only increased in upper leaves 14 DAF. The well-known regulation of NAC transcript abundance by miR164 in Arabidopsis (Guo et al., 2005; Kim et al., 2009) prompted us to examine whether it could also apply to pea. By exploiting an internal miRNA database, we observed that NAC1 and NAC100 are indeed predicted targets of members of the miR164 family in pea (Table S6).

### TF-Related Co-expression Modules in Pea Leaves

To predict putative regulations by the TFs, a co-expression network based on high Pearson correlations (*r* <-0.95 or >0.95) was built from the normalized intensities (Log_2_) of the 48 samples hybridized on the arrays. Variables with low overall variance were filtered out to reduce the impact of noise (see Materials and Methods). The filtered dataset (11949 probes), provided in Table S7, can be imported in Cytoscape and easily converted into an interaction Network using the Expression Correlation package (Cline et al., 2007). This Pea REMObilization NETwork (P-REMONET) consisted of 4523 nodes (i.e., genes) and 67447 edges (i.e., co-expression links). A total of 436 components were identified in P-REMONET, the largest containing 3225 nodes/genes (Figure S3A). Of the TF genes differentially regulated at least 4-fold, 39 were connected to one, two, or many genes. Several TFs were linked together, leading to 30 different TF-related modules (Table 1). The list of genes in each module is available in Table S8 along with the strength (r), type of interaction (i.e., correlation either positive or negative), and expression patterns. Several modules contain TF genes whose regulation depends on nitrogen availability, such as NAC073#2 and NAC043, which were down-regulated 27 DAF specifically under nitrate deficiency (Table 1 and Table S8).

**Table 1:** TF-related modules in the P-REMONET co-expression network.

**TFs in Figure 4B (differentially regulated by at least 4-fold between stages)**	**Cluster (Figure 4B)**	**Nodes pos-neg**	**Edges**	**% genes regulated by N**	**Module (ID)**	**Additional TF sequences in the module (regulated <4-fold between stages)**
PsCam039498_ERF/AP2#1^*†*^	VI	192-4	7694	25%	M1 (197)	PLATZ (PsCam038319)^†^ ERF/AP2#3 (PsCam039388)
PsCam040607_bHLH#1	VI	69-0	1465	20%	M2 (70)	
PsCam037583_BZIP61	VI	63-0	1338	3%	M3 (64)	GATA-type zinc finger (PsCam020847) bHLH#2 (PsCam052606)
PsCam038898_MYB83^*†*^	VI	38-3	264	33%	M4 (89)	PLATZ (PsCam038319)^*†*^
PsCam038865_MYB46	VI	58-2	1064	20%		
PsCam056008_bHLH70	VI	44-14	679	27%	M5 (59)	Myb12 (PsCam009746) bZIP34 (PsCam037351) ERF/AP2#3 (PsCam039388)
PsCam037387_MYB55#3	VI	40-0	339	30%	M6 (102)	RING/FYVE/PHD-type znf (PsCam038341)^†^ GATA9 (PsCam039673)
PsCam052042_IAA14, SLR	VI	21-0	122	14%		
PsCam027699_GATA4^*†*^	VI	48-0	630	30%		
PsCam003183_KDR/bHLH#3	VI	48-0	726	16%		
PsCam037889_NAC073#1	VI	40-0	413	41%	M7 (50)	NAC043 (PsCam000593)^†^
PsCam006892_NAC073#2^*†*^	VI	38-0	388	49%		
PsCam038695_MYB55#1	VI	4-0	5	25%	M9 (19)	–
PsCam010929_MYB55#2^*†*^	VI	15-0	58	44%		
PsCam002187_IAA19, MSG2^*†*^	VI	6-0	15	43%	M14 (7)	–
PsCam002893 MIF3	VI	13-2	44	37%	M13 (24)	–
PsCam043483_MYB-like^†^	VI	9-2	35	67%		
PsCam033601_NAC2, NAC056	I	1-9	33	54%		
PsCam038037_NAC100	I	9-3	31	54%	M10 (13)	–
PsCam001138_WRKY23	I	0-3	3	75%	M20 (4)	–
PsCam048143_MYB78^†^	I	0-1	1	50%	M27 (2)	–
PsCam046167_HSF4	II	3-0	3	25%	M19 (4)	–
PsCam051031_Homeobox 7^†^	II	1-0	1	100%	M24 (2)	–
PsCam003292_WRKY75	II	1-0	1	0%	M26 (2)	–
PsCam005232_MYB48#1^*†*^	IV	2-24	306	37%	M8 (27)	–
PsCam011255_MYB48#2^*†*^	IV	4-1	8	83%	M15 (6)	–
PsCam036109_Homeodomain-like	IV	4-0	5	20%	M16 (5)	–
PsCam029461_NAC029	IV	3-1	6	80%	M17 (5)	–
PsCam034575_AGAMOUS-like 8^†^	IV	1-0	1	50%	M28 (2)	–
PsCam005456_AGAMOUS-like 6	V	0-1	1	0%	M29 (2)	–
PsCam055941_NF-YA3^†^	V	10-0	32	81%	M11 (11)	–
PsCam025011_MYB63^†^	VII	10-0	40	9%	M12 (11)	Zinc finger-type (PsCam004767)
PsCam002076_ ERF/AP2#2^*†*^	VII	2-0	2	0%	M21 (3)	ERF/AP2#4 (PsCam039693)
PsCam048251_MYC2^*†*^	VII	1-0	1	100%	M22 (2)	JAZ5 (PsCam001382)^†^
PsCam002359_RRTF1	VII	1-0	1	50%	M23 (2)	–
PsCam002549_Integrase-type^†^	VII	1-0	1	100%	M25 (2)	Integrase-type DDF1 (PsCam002503)^†^
PsCam036217_WRKY41	VII	1-0	1	50%	M30 (2)	–
PsCam040360_Dof-type	VII	3-0	4	25%	M18 (4)	–

The table describes the co-expression modules containing the TFs differentially expressed at least 4-fold in leaves between two developmental stages. The modules were retrieved from P-REMONET (Figure S3A).

† *indicates that gene expression varied significantly in response to nitrate (N) nutrition. The cluster in Figure 4B to which belong the TFs is indicated, along with the number of positive (pos) and negative (neg) connections, of edges, proportion of genes regulated by nitrogen availability, module, and number of different IDs/genes in the module. In the last column are additional TFs, regulated <4-fold, in the modules. Details about genes in each module are provided in Table S8*.

To investigate the robustness of P-REMONET for predicting TF-TF or TF-target interactions, a search for the best Arabidopsis homologs was performed for each gene in the TF-related modules. The P-REMONET predictions showed similarities to interactions validated in Arabidopsis. For example, module M22 consisted of two positively correlated genes, PsCam002187 and PsCam001382, respectively, homologous to MYC2 and JAZ5 (jasmonate-zim-domain protein 5), which interact in yeast two-hybrid assays (Chini et al., 2009). Module M4 was enriched for genes related to cell wall biosynthesis and contains two potential regulators, MYB46 (PsCam038865) and MYB83 (PsCam038898), shown in Arabidopsis to bind to the same secondary wall MYB-responsive element consensus sequence and activate the same set of direct targets involved in secondary wall biosynthesis (Zhong and Ye, 2012). Module M7 for two NAC073 TFs sharing 70% homologies (NAC073#1 and NAC073#2) was enriched in genes for cellulose biosynthesis, including two cellulose synthase genes. Consistently, NAC073 in Arabidopsis was named SND2 for Secondary wall-associated NAC Domain protein 2 and transactivates the cellulose synthase 8 promoter (Hussey et al., 2011). These observations validated P-REMONET as a useful tool to predict relevant regulations.

The largest TF-related modules in P-REMONET contain genes down-regulated during the time course (TFs belonging to cluster VI in Table 1). The higher number of connections was identified for module M1, which contained 197 genes connected to the ethylene response factor/Apetala2 TF (ERF/AP2#1, PsCam039498, Table 2), suggesting this TF acts as a hub. Several TFs in these modules could act in concert since they were positively connected: ERF/AP2#1 and a plant AT-rich sequence and zinc-binding protein (PLATZ) in module M1, bZIP61, bHLH#2, and a GATA-type zinc finger TF in module M3, bZIP34, bHLH70, MYB12, and ERF/AP2#3 in module M5 (Table 1). An analysis of GO terms for the co-expressed genes predicted biological processes that could be repressed in coordination with the down-regulation of the TFs (Table S8).

**Table 2:** TF-related co-expression modules conserved between pea and *M. truncatula*.

**Pea TF ID clusters in Figure 4B modules in Table S8**	***M. truncatula* ID**	**Pea sequence ID of genes connected to the TFs**	**Best *M. truncatula* homologs also connected to the TFs**	**Gene annotation (best Arabidopsis homolog)**
**ERF/AP2^#^1**† PsCam039498 Cluster VI, **module M1** (down-reg. 14 DAF in lower and upper leaves)	MT0007_00880	PsCam049838 (+) PsCam038319 (+) PsCam025580 (+) PsCam023684 (+) PsCam036606 (+) PsCam012843 (+) PsCam036120 (+) PsCam027004 (+)	MT0011_00523 (+) MT0031_10256 (+) MT0003_11081 (+) MT0003_11081 (+) MT0040_10268 (+) MT0031_00200 (+) MT0067_10073 (+) MT0010_00419 (+)	Protein kinase (AT3G26700) **PLATZ transcription factor** (AT1G32700) Extensin-like; proline-rich cell wall protein (AT4g38770) Extensin-like; proline-rich cell wall protein (AT4g38770) SKU5, cell wall modifying enzyme (AT1G76160) *S*-adenosylmethionine synthetase (AT2G36880) Invertase/pectin methylesterase inhibitor (AT4G02320) Glycosyl hydrolase 9B8 (AT2G32990)
				
**MYB83**† PsCam038898 Cluster VI, **module M4** (down-reg. 14 DAF in lower and upper leaves)	MT0003_10639	PsCam012843 (+) PsCam036606 (+) PsCam038319 (+) PsCam006765 (+) PsCam005256 (+)	MT0031_00200 (+) MT0040_10268 (+) MT0031_10256 (+) MT0066_10032 (+) MT0012_10060 (+)	*S*-adenosylmethionine synthetase (AT2G36880) SKU5, cell wall modifying enzyme (AT1G76160) **PLATZ transcription factor** (AT1G32700/AT4G17900) Laccase 17 (AT5G60020) Adenine nucleotide alpha hydrolases-like (AT2G03720)
				
**bHLH70**PsCam056008 Cluster VI, **module M5** (down-reg. 14 DAF in upper leaves)	MT0028_10309	PsCam034290 (−)	MT0002_10493 (−)	Weak chloroplast movement under blue light-like protein (DUF827) (AT2G26570)
				
**NAC073^#^1** PsCam037889 Cluster VI, **module M7** (down-reg. 14 DAF in lower and upper leaves and 27 DAF in upper leaves under nitrate deficiency)	MT0019_00537	PsCam038807 (+) PsCam043546 (+) PsCam033940 (+) PsCam057773 (+) PsCam036683 (+) PsCam000957 (+) PsCam023534 (+) PsCam013191 (+)	MT0003_00396 (+) MT0001_01114 (+) MT0001_00233 (+) MT0010_00304 (+) MT0040_10197 (+) MT0039_00275 (+) MT0039_00275 (+) MT0039_00275 (+)	FASCICLIN-like arabinogalactan-protein 12 (AT5G60490) Protein of unknown function, DUF538 (AT2G03350) Cellulose synthase CESA7 (AT5G17420) TRICHOME BIREFRINGENCE-LIKE 33 (AT2G40320) Glycosyl hydrolase 9B5 (AT1G19940) GERMIN-LIKE, GLP10 (Cell wall-related, AT3G62020) GERMIN-LIKE, GLP10 (Cell wall-related, AT3G62020) GERMIN-LIKE, GLP10 (Cell wall-related, AT3G62020)

The pea sequence IDs were from the Pea Gene Atlas (http://bios.dijon.inra.fr/) and the M. truncatula IDs are from the Symbimics program (https://iant.toulouse.inra.fr/symbimics/). The best M. truncatula homologs (v4.02) were identified using OrthoFinder v1.1.8. The signs indicate whether the correlation with TF gene expression was positive (+) or negative (–). Genes were annotated by homology with sequences in The Arabidopsis Information Resource (TAIR, https://www.arabidopsis.org/).

# *indicates that gene expression varied significantly in response to nitrate nutrition*.

**Figure 5:**
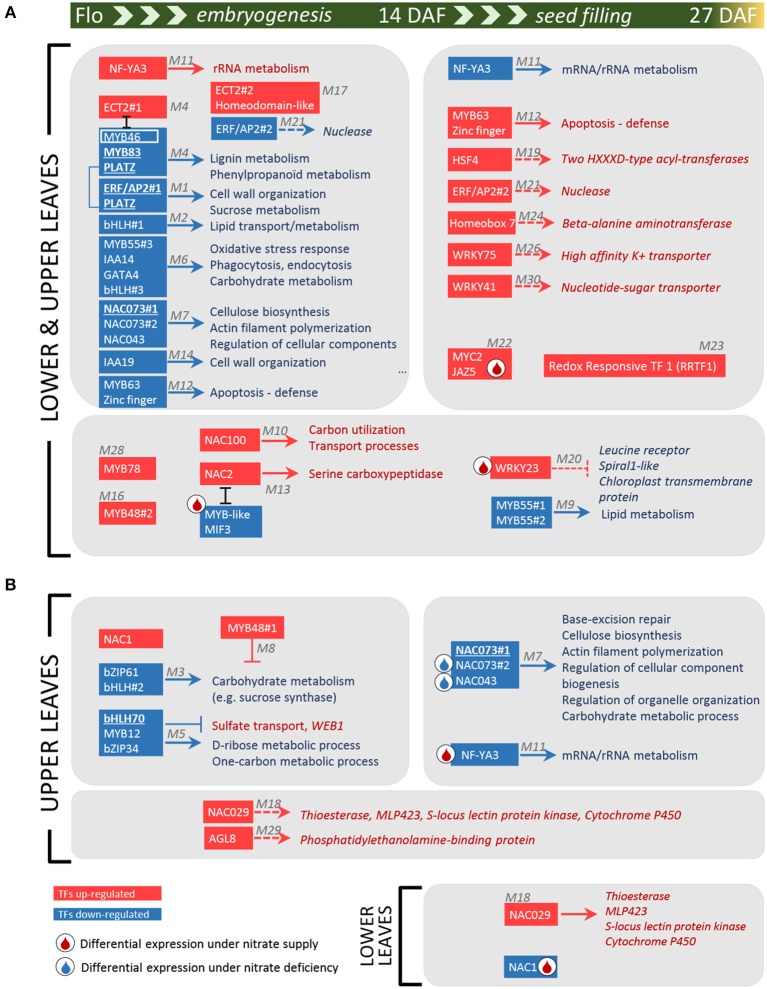
Diagram of TF-related co-expression modules in pea leaves during embryogenesis and seed filling. Red and blue rectangles correspond to TFs up- and down-regulated, respectively, during the time course of nutrient remobilization: from the beginning of flowering to 14 DAF (left panel), from 14 to 27 DAF (right panel) or throughout the time course (large panels). The arrows indicate a co-expression between TFs and biological processes (TopGO annotation, Table S8) or genes (in italic) was identified in P-REMONET (corresponding modules in gray). ⊥ and **エ** indicate negative expression correlations between genes. The colors of the TFs and related processes indicate the genes were up- (red) or down- (blue) regulated during the time course in both lower and upper leaves **(A)** or in specific leaf types **(B)**. TFs in bold and underlined have shared putative targets in the co-expression networks from *M. truncatula* and pea. A droplet indicates significant variations in gene expression specifically in response to nitrate supply (red) or deficiency (blue).

Other TFs were positively connected with genes up-regulated at 14 or 27 DAF, thus identifying some putative transcriptional activators of processes induced during the time course (Table 1 and Table S8). Two of these modules, M11 and M12, are depicted in Figures S3B,C since they contain the higher number of positive links with the TFs. The TF in module M11 (PsCam055941) was homologous to the subunit A3 of the nuclear factor Y (NF-YA3, AT1G72830), which in Arabidopsis stimulates the transcription of various genes by recognizing and binding to a CCAAT motif in promoter regions (Leyva-González et al., 2012). In pea, *NF-YA3* was up-regulated in lower and upper leaves 14 DAF compared to flowering, then down-regulated 27 DAF (cluster V in Figure 4B), highlighting important regulations of this gene during the time course investigated. In contrast, the TF gene in module M12 (PsCam025011), homologous to *MYB63* (AT1G79180), was down-regulated 14 DAF, then up-regulated 27 DAF in both vegetative and upper leaves (cluster VII in Figure 4B), suggesting a role at the transition stage toward chlorophyll breakdown and senescence. Annotations of the co-expressed genes indicated that MYB63 may activate defense responses. These data were summarized in Figure 5, which provides a global view of the TF-related co-expression modules identified in pea leaves, depending on the developmental stages and nitrate availability.

### Comparing Nitrogen Remobilization and TF Modules Between Pea and *M. truncatula*

**Figure 6:**
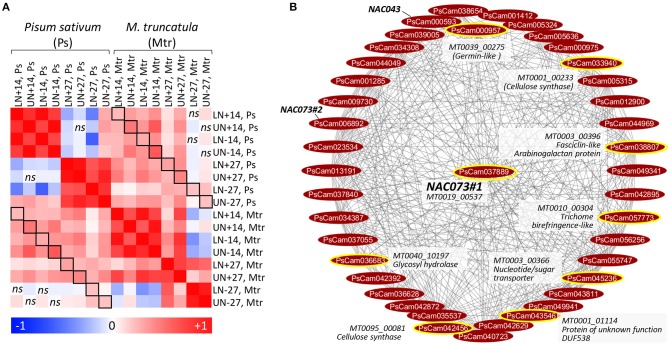
Comparison of leaf transcriptomes and TF-related modules between pea and *M. truncatula*. **(A)** Pair-wise Pearson correlation coefficients calculated from each sample comparison expressed in log_2_ ratio: beginning of flowering vs. 14 DAF (14), 14 vs. 27 DAF (27), for lower (L), and upper (U) leaves, with nitrogen (N+), or without nitrogen (N–). The color scale indicates the degree of correlations between transcriptomes. All correlations were significant (*P* < 0.05, *n* = 14980 sequences) except sample comparisons indicated by *ns* (non-significant). Squares indicate the same samples compared between the two species. **(B)** NAC073#1 (PsCam037889)-related network in module M7. The connected genes were organized using the circular layout algorithm from Cytoscape. Orthologous genes connected to the NAC073#1 ortholog (MT0019_00537) in *M. truncatula* (data from Table 2) were encircled in yellow and annotated.

A comparative study in *M. truncatula* was performed by coupling nitrogen remobilization analysis at the whole plant level with a transcriptome analysis of leaf samples collected under the same conditions as were the pea samples. The dynamic of nitrogen remobilization was similar between pea and *M. truncatula* from flowering to 14 DAF (Figure S4). Some differences occurred between 14 and 27 DAF: unlike pea, nitrogen was mainly remobilized from lower leaves of *M. truncatula* during this period. For transcriptomics comparisons, we focused on the 14980 orthologous sequences with a unique gene per species. A Pearson's distance correlation matrix was generated to compare transcriptomics data (expressed in log_2_ ratio) between pea and *M. truncatula* (Figure 6A). The correlations were positive between species (0.13≤r≤0.45) for all pairwise comparisons, indicating transcriptional regulations at least in part conserved between the species.

To identify shared regulators between pea and *M. truncatula*, we focused on the 39 TF genes highly regulated in pea leaves and for which putative targets were identified in P-REMONET. A *M. truncatula* ortholog was found for 31 of these TFs (Table S9). After building a gene co-expression network (M-REMONET) from the normalized intensities (Log_2_) of the 48 leaf samples hybridized on the *M. truncatula* arrays (21164 probes, 8778 nodes, 108210 edges), a search for co-expression modules containing these TFs was performed. Four TFs (ERF/AP2#1, MYB83, bHLH70, NAC073#1) were closely connected to genes orthologous between the species (Figure 5). These putative conserved targets were listed in Table 2 along with the type of correlation with the TFs (positive or negative). Notably, the connected genes in modules M1, M4, and M7 (Table 2) were related to cell wall metabolism/structure, suggesting important transcriptional regulation of cell wall structure in leaves of both species. In the M7 module depicted in Figure 6B, of the eight genes similarly connected to NAC073#1 in both species, seven were related to cell wall metabolism (Table 2). Almost all genes in this module were down-regulated in lower and upper leaves 14 DAF (Table S8), suggesting major modifications of cell wall structure in these leaves at early reproductive stages.

## Discussion

To provide a first overview of the transcriptional regulations occurring in pea leaves during seed development, we focused on stages of the reproductive phase preceding the sharp decrease in chlorophyll breakdown, up to a transition stage toward senescence (27 DAF, Figure 1). A long-term ^15^N-nitrate-labeling experiment indicated that these stages were associated with dynamic nitrogen recycling and remobilization between tissues, leaves from vegetative and reproductive nodes contributing, respectively, to 29 and 44% of the total amount of nitrogen remobilized during this period (Figure 1). The subsequent stages were associated with nitrogen recycling from all tissues, including pod walls, and at maturity, 54% of nitrogen accumulated in pea seeds was derived from remobilization processes (Figure 1). Our data demonstrated that leaves are the main source of remobilized nitrogen, followed by pod wall, roots and stems, which is consistent with data previously obtained in a pulse-chase ^15^N-labeling experiment (Schiltz et al., 2005). A transcriptome analysis of leaves from vegetative and reproductive nodes from flowering to 27 DAF showed that most of the well-known SAG, such as the cysteine protease gene *SAG12*, were not significantly up-regulated in our leaf samples. By contrast, genes that might contribute to promote nutrient recycling while maintaining leaves in a healthy metabolic state, i.e., with limited protein degradation, were identified. Complemented by a gene co-expression approach targeted on the most regulated TFs, this study provides a repertoire of regulatory predictions, some of which were conserved in the forage legume species *M. truncatula* (Table 2), that can broadly serve as a backdrop for studying the role of individual genes in legumes.

### Molecular Features of Leaves From the Reproductive Nodes During Seed Embryogenesis

#### Sulfur Transport and Metabolism

From the beginning of flowering to 1st node seed filling (14 DAF), seeds progress through embryogenesis on the reproductive nodes. This period was associated with deep transcriptional changes in leaves of the reproductive nodes regardless of nitrate availability (Figure 2A), as exemplified by transporter gene expression (Figure 3A and Table S4). *SULTR* genes were among the most up-regulated transporter genes in upper pea leaves 14 DAF as compared to flowering (Figure 3A). The most up-regulated was homologous to SULTR2;1 (PsCam025051, Figure 3C), which has been shown in Arabidopsis to be expressed in vascular tissues and proposed to regulate internal translocation and distribution of sulfate (Takahashi et al., 2000). The over-representation of genes related to methionine metabolism in the set of genes up-regulated 14 DAF in leaves of the reproductive nodes suggests sulfate can be used for methionine metabolism in these leaves (Figure 2A). Sulfate transport in upper pea leaves can also contribute to avoid precocious senescence owing to the role of sulfate-derived compounds in preventing autophagy and senescence in Arabidopsis and tomato (Álvarez et al., 2012; Yarmolinsky et al., 2014).

The gene co-expression approach enabled us to deduce some possible regulators of SULTR2;1/PsCam025051. The transporter was positively connected to five genes in P-REMONET, one of which encodes a Ser/Thr kinase (PsCam034543, Figure 3D). In the green alga *Chlamydomonas reinhardtii*, a Snf1-like Ser/Thr kinase positively regulates sulfate transporters (Davies et al., 1999), and in Arabidopsis all the substitutions at the phosphorylation site Thr-587 of a SULTR led to a complete loss of sulfate transport (Rouached et al., 2005). Hence, the Ser/Thr kinase may be a promising candidate for investigating the signal transduction system regulating sulfate homeostasis in upper leaves. In addition, SULTR2;1/PsCam025051 was negatively connected to two TF genes homologous to *bHLH70* and *MYB12* (module M5 and Figure 3D). Many MYB/bHLH complexes have been described in plants (Pireyre and Burow, 2015) and MYB factors have been shown to regulate genes related to sulfate assimilation (Koprivova and Kopriva, 2014), reinforcing the interest of further studies on the interplay of these genes.

#### Other TF Candidates for Maintaining Leaf Metabolism or Preventing Senescence

The above-mentioned *bHLH70* gene was among the most down-regulated TFs 14 DAF (cluster VI in Figure 4B). It was connected to genes with different functions in module M5, suggesting pleiotropic roles. In particular, *bHLH70* was negatively connected to *WEB1* (weak chloroplast movement under blue light 1) in both P- and M-REMONETs (Table 2), pointing out bHLH70 as a putative repressor of *WEB1* expression in leaves of both forage and grain legume species. WEB proteins maintain the velocity of chloroplast movements *via* chloroplast-actin filaments in response to ambient light conditions (Kodama et al., 2010). By controlling chloroplast redistribution, they prevent the dismantling of the photosynthetic apparatus by excess light. The increased *WEB1* expression in upper leaves at 14 DAF may be part of the mechanisms by which photosynthesis is maintained before senescence initiation. Our data suggest bHLHL70 to be a good candidate for investigating the regulation of these mechanisms. Another TF candidate up-regulated in upper pea leaves at 14 DAF is *NAC1/PsCam050102* (Figure 4B). Overexpression of a NAC1-type TF in wheat delayed leaf senescence, leading to a stay-green phenotype (Zhao et al., 2015). Therefore, the up-regulation of *NAC1* in upper leaves could contribute to prevent senescence, even when nitrate absorption by roots becomes limiting (Figure 1). The mRNA abundance of NACs, including NAC1, is controlled by miR164 in Arabidopsis^25^. It was therefore interesting to observe that all four predicted targets of miR164 in pea belong to the NAC family (Table S6), of which one corresponds to NAC1. This reinforces the possible regulation of NAC1 transcript abundance by miR164 in pea leaves.

### The Early Reproductive Phase Is Accompanied by a Reprogramming of Cell Wall-Related Genes in Leaves of Both Vegetative and Reproductive Nodes

Genes of lignin catabolism and cell wall organization were enriched in the list of genes down-regulated 14 DAF in lower and upper pea leaves (Figure 2A), reflecting a shift in cell wall structure at early reproductive stages. Interestingly, three TF-related modules conserved between pea and *M. truncatula* contained genes of cell wall metabolism/organization (Table 2). These conserved modules, described in Table 2, were identified for:

(*i*) ERF/AP2#1, which shares homologies with Arabidopsis AP2 TFs that have roles in plant protective layers such as the cuticle (Aharoni et al., 2004). In pea and *M. truncatula, ERF/AP2#1* was positively linked to five genes related to cell wall organization and to a PLATZ TF responsible for A/T-rich sequence-mediated transcriptional repression (Nagano et al., 2001). The identification of PLATZ and ERF/AP2 in the cell wall network built from a co-expression analysis in rice (Hirano et al., 2013) reinforces their possible coordinated function in controlling cell wall structure.

(*ii*) MYB83, which was similarly connected to the PLATZ TF and co-expressed with a gene encoding an oxidative enzyme (laccase, *LAC17*, Table 2) proposed to determine the pattern of cell wall lignification (Schuetz et al., 2014). The role of MYB83 in secondary wall biosynthesis has been demonstrated in Arabidopsis, where its overexpression induced the expression of secondary wall biosynthetic genes and resulted in an ectopic deposition of secondary wall components. In P-REMONET, MYB83 was linked to a third TF, MYB46 (module M4 in Table S8), shown in Arabidopsis to act redundantly with MYB83 in regulating secondary cell wall biosynthesis (McCarthy et al., 2009). The authors have shown that simultaneous RNAi inhibition of MYB83 and MYB46 reduced secondary wall thickness in fibers and vessels. Other authors demonstrated that MYB46 was sufficient to induce the entire secondary wall biosynthetic program (Zhong et al., 2007).

(*iii*) NAC073#1, which was positively linked to eight genes orthologous between pea and *M. truncatula*, of which five may have roles in cell wall formation/organization (2 cellulose synthases, a trichome birefringence-like protein, a glycosyl hydrolase and a Fasciclin-like Arabinogalactan protein, Figure 6B). In pea, NAC073#1 was positively connected to two additional NAC TFs: NAC073#2 and NAC043 (also named NST1 for Secondary Wall Thickening Promoting Factor1) (Figure 6B). Evidence is accumulating to suggest that a subset of closely related NACs act as master transcriptional switches governing secondary wall biosynthesis and fiber development (Zhong et al., 2008). In Arabidopsis, NAC073 and NAC043/NST1 contribute to the formation of secondary cell wall, and their repression resulted in a remarkable reduction in the secondary wall thickening (Zhong et al., 2008).

Taken altogether, the data indicate that the transcriptional regulation of cell wall organization and metabolism in leaves of legumes occurs at early reproductive stages and may involve seven transcription factors pinpointed here for the first time in pea: ERF/AP2#1, a PLATZ TF, MYB83, MYB46, NAC073#1, NAC073#2, and NAC043. The expression of *NAC073#2* and *NAC043* decreased 27 DAF under nitrate-deficiency only (Figure 5B), indicating that the intricate control of cell wall metabolism in pea leaves may rely on nitrate-dependent regulations. Although data accumulate in the literature on the role of NAC TFs in regulating cell wall metabolism (Zhong et al., 2007, 2008; McCarthy et al., 2009; Hirano et al., 2013; Schuetz et al., 2014), the full list of their targets remains to be established. The present study highlighted some putative targets for further investigations (Figure 6B).

### Transcriptional Reprogramming of Leaves at a Transition Stage Toward Senescence

Transcriptome changes in pea leaves 27 DAF, which marks the switch toward senescence-associated yellowing (Figure S1), contributed to our understanding of molecular events underlying this transition.

#### Autophagy-Related Processes

GO enrichment analysis of genes up-regulated in leaves 27 DAF revealed an over-representation of genes involved in defense responses, such as disease resistance proteins (R proteins, Figure 2B). Accordingly, several defense-related genes known to be induced by pathogens were found to be expressed during Arabidopsis leaf senescence in a pathogen-independent manner (Quirino et al., 1999). Seven R protein genes up-regulated 27 DAF were in the MYB63-related module (M12 in Figure S3C). All contain an NB-ARC domain (Nucleotide-Binding adaptor shared by Apoptotic protease-activating factor-1, R proteins, and *Caenorhabditis elegans* death-4 protein) essential for protein activity (van Ooijen et al., 2008; Table S10). Interestingly, in rice, an R protein with NB-ARC domain has been named RLS1 (Rapid Leaf Senescence 1) because the disruption of the gene accelerated leaf senescence due to a rapid loss of chlorophyll (Jiao et al., 2012). The authors showed that *RLS1* is involved in the autophagy-like programmed cell death and partial degradation of chloroplast. The R proteins in module M12 could play a similar role in the autophagy-mediated programmed cell death to promote nutrient remobilization while avoiding rapid senescence. By activating the autophagy process, reactive oxygen species (ROS) are key players in the regulation of programmed cell death (Pérez-Pérez et al., 2012). Therefore, the increased expression 27 DAF of *RRTF1* (Figure 5A, module M23 in Table S8), encoding the Redox-Responsive TF1 that controls positively the accumulation of ROS in Arabidopsis shoots and roots (Matsuo et al., 2015), might contribute to orchestrate autophagy-mediated programmed cell death. Because autophagy allows the remobilization of nutrients while preserving cell longevity, identifying autophagy regulators is of particular interest. In module M12, all R proteins were positively connected to *MYB63*, which plays a dual function in regulating secondary cell wall formation and genes involved in disease resistance in Arabidopsis (Zhou et al., 2009). MYB63 and three R proteins were also positively connected to a Zinc finger-type TF whose closest Arabidopsis homolog (AT2G40140) was ROS-responsive (Gadjev et al., 2006). All these features indicate these two TFs may regulate autophagy, possibly through ROS perception.

#### Transporters

In the quest to identify transporters contributing to the recycling of nutrients at the transition toward senescence, the list of transporter genes differentially regulated during the developmental period was examined (Table S4). The most up-regulated nitrogen transporters were high-affinity transporters of basic amino acids (e.g., CAT5) and nitrate (NRT2.5). In Arabidopsis, NRT2.5 plays a role in nitrate loading into the phloem during remobilization processes under nitrogen starvation (Lezhneva et al., 2014). The *NRT2.5* homolog in pea was up-regulated 27 DAF in lower and upper leaves whatever nitrate supply (Table S4), suggesting a contribution to nitrogen recycling not restricted to low nitrate environments in pea. Although a role for CAT5 in leaf nitrogen remobilization has not yet been demonstrated, one *CAT5* gene (At2g34960) was up-regulated in senescing Arabidopsis leaves (van der Graaff et al., 2006). The up-regulation of *CAT5* in both leaf types 14 DAF and specifically in response to nitrate-deficiency 27 DAF (Table S4) suggests this gene could contribute to the recycling of amino acids in pea leaves, notably under nitrate-deficiency at later stages. However, nitrogen/amino acid transporters were not among the most regulated genes at 27 DAF, contrarily to genes encoding transporters of nucleotides, sugars, lipids, phosphate, potassium, nickel, and copper, which were up-regulated at least 4-fold at this stage (Table S4). Although these genes have not been reported to play a role in preventing rapid senescence, potassium homeostasis is known to play an essential role in stress-induced senescence (Anschütz et al., 2014), and a recent study highlighted the need to maintain potassium levels in leaves during nitrate starvation to prevent senescence (Meng et al., 2016). The potassium transporter identified (PsCam042603) was homologous to the high affinity K+ transporter gene *HAK5*. In P-REMONET, HAK5 was connected to a TF gene homologous to *WRKY75* (Figure 6A), which has been shown to be induced during potassium starvation in Arabidopsis (Devaiah et al., 2007), highlighting the interest to investigate the relationship between this TF and the regulation of potassium transport in leaves.

#### Translation-Associated Processes

By influencing ribosome structure and function, ribosomal RNA (rRNA) processing, and modifications play key roles in protein synthesis, and thereby control metabolic activities (Bohne, 2014). Interestingly, genes of rRNA processing and modifications, and of translation were among the most represented in the list of genes down-regulated 27 DAF, compared to 14 DAF, in lower leaves, especially under nitrate-deficient conditions (Figure 2B). This suggests reduced translational activities in these leaves at the transition toward senescence. Genes related to these functional categories were among the most over-represented in the set of genes up-regulated 14 DAF, compared to flowering, in lower and upper pea leaves (Figure 2A), emphasizing the importance of these processes at early reproductive stages. The relationship between these genes and the progression toward senescence in leaves has not yet been established. However, perturbations of rRNA biogenesis are closely related to cell senescence in human cells (Yuan et al., 2017). Importantly, six of these genes were positively connected to the *NF-YA3* TF (Figure S3B and Table S8), making it a good candidate for controlling metabolic activities in leaves. In Arabidopsis, overexpression of *NF-YA* members resulted in dwarf late-senescing plants (Leyva-González et al., 2012). Furthermore, overexpression of the soybean gene *NF-YA3* in Arabidopsis enhanced drought resistance (Ni et al., 2013), indicating this nuclear factor subunit may be associated with protective roles in plants, but the targets potentially co-regulated by the NF-Y complex are yet to be identified. Our results pinpoint genes in module M11 (Table S8) as attractive candidates for a deeper study of NF-YA3 function in leaves.

Overall, our results provided new information in understanding the complexity of the transcriptional regulations governing leaf metabolism during seed development in pea up to the transition toward senescence. These findings could serve future in depth investigations on specific genes or TF-related modules.

## Data Availability

The datasets generated for this study can be found in NCBI Gene Expression Omnibus database, GSE109789 for pea and GSE109521 for M. truncatula.

## Author Contributions

JB conceived the project. JB, GA, and SB conceived the pea 40 k-arrays. KG, AK, CS, and AL designed the nitrogen remobilization experiment, and the overall research plan with JB. AB contributed to all experiments with GA and MS (molecular aspects), AK and CL (phenotyping and greenhouse experiments), SP and SB (microarray analyses). KG developed the gene networks with contribution of CH and built the nitrogen remobilization diagrams with contributions of JT and J-CA. MT provided the miR164 data and performed the othology search between species. KG analyzed all the data, with contribution of AB for phenotypic characteristics, and wrote the manuscript.

### Conflict of Interest Statement

The authors declare that the research was conducted in the absence of any commercial or financial relationships that could be construed as a potential conflict of interest.
