# Supplementary material for: Transcriptional Reprogramming of Pea Leaves at Early Reproductive Stages
Source: Front Plant Sci. 2019 Aug 7;10:1014. doi: 10.3389/fpls.2019.01014 (PMC6693388; doi:10.3389/fpls.2019.01014)
Supplement: Supplementary file 2 [file Presentation_1.PPTX]

## Slide 1
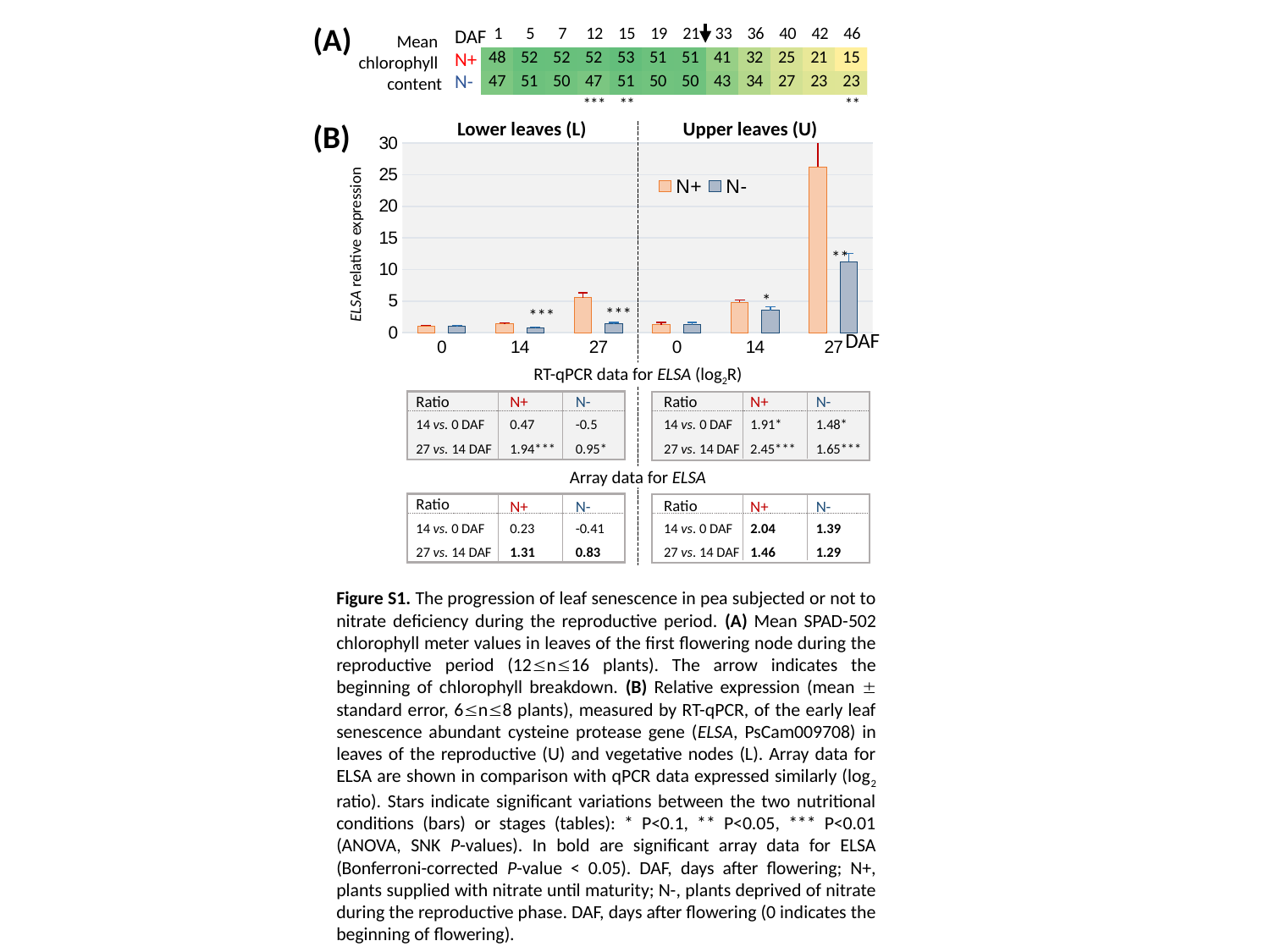

(A)
DAF
Mean
chlorophyll
content
N+
N-
Lower leaves (L)
Upper leaves (U)
(B)
### Chart
| Category | N+ | N- |
|---|---|---|
| 0 | 1.0381028300249187 | 1.0381028300249189 |
| 14 | 1.4373439016690974 | 0.7188601766514543 |
| 27 | 5.529218273289978 | 1.3865317502794168 |
| 0 | 1.27375834512388 | 1.27375834512388 |
| 14 | 4.80833428838355 | 3.5637246083525302 |
| 27 | 26.240388334519565 | 11.223232587652344 |ELSA relative expression
**
*
***
***
DAF
RT-qPCR data for ELSA (log2R)
Ratio
N+
N-
Ratio
N+
N-
14 vs. 0 DAF
0.47
-0.5
14 vs. 0 DAF
1.91*
1.48*
27 vs. 14 DAF
1.94***
0.95*
27 vs. 14 DAF
2.45***
1.65***
Array data for ELSA
Ratio
Ratio
N+
N-
N+
N-
14 vs. 0 DAF
0.23
-0.41
14 vs. 0 DAF
2.04
1.39
27 vs. 14 DAF
1.31
0.83
27 vs. 14 DAF
1.46
1.29
Figure S1. The progression of leaf senescence in pea subjected or not to nitrate deficiency during the reproductive period. (A) Mean SPAD-502 chlorophyll meter values in leaves of the first flowering node during the reproductive period (12n≤16 plants). The arrow indicates the beginning of chlorophyll breakdown. (B) Relative expression (mean ± standard error, 6n≤8 plants), measured by RT-qPCR, of the early leaf senescence abundant cysteine protease gene (ELSA, PsCam009708) in leaves of the reproductive (U) and vegetative nodes (L). Array data for ELSA are shown in comparison with qPCR data expressed similarly (log2 ratio). Stars indicate significant variations between the two nutritional conditions (bars) or stages (tables): * P<0.1, ** P<0.05, *** P<0.01 (ANOVA, SNK P-values). In bold are significant array data for ELSA (Bonferroni-corrected P-value < 0.05). DAF, days after flowering; N+, plants supplied with nitrate until maturity; N-, plants deprived of nitrate during the reproductive phase. DAF, days after flowering (0 indicates the beginning of flowering).

## Slide 2
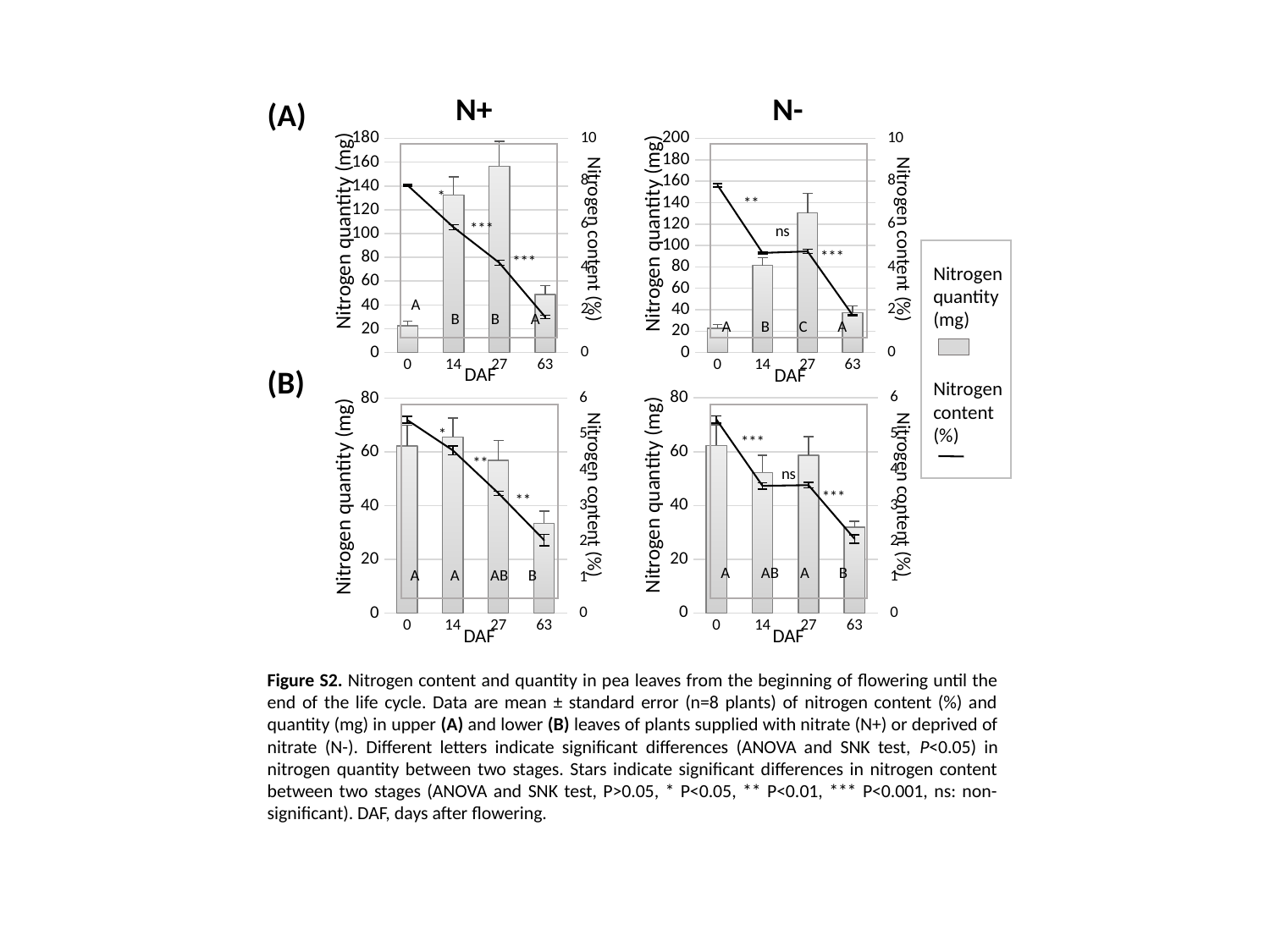

N+
N-
(A)
### Chart
| Category | Q N (mg) | %N |
|---|---|---|
| 0 | 22.57967548155885 | 7.80965861878387 |
| 14 | 81.28152674849642 | 4.65070458730615 |
| 27 | 130.42846228948778 | 4.72222563400737 |
| 63 | 37.05747170457448 | 1.75008868891772 |
### Chart
| Category | Q N (mg) | %N |
|---|---|---|
| 0 | 22.57967548155885 | 7.80965861878387 |
| 14 | 132.49825888006728 | 5.85343076869002 |
| 27 | 156.65594132685936 | 4.19213629818457 |
| 63 | 48.62419155490845 | 1.66484144128562 |
*
**
Nitrogen quantity (mg)
***
Nitrogen quantity (mg)
ns
***
***
Nitrogen content (%)
Nitrogen content (%)
Nitrogen quantity (mg)
A
B
B
A
A
B
C
A
DAF
(B)
DAF
Nitrogen content
(%)
### Chart
| Category | DW | Q N (mg) |
|---|---|---|
| 0 | 62.33103702989848 | 5.39726911471266 |
| 14 | 52.29334710734694 | 3.54855950241556 |
| 27 | 58.595670164343325 | 3.5671427366964 |
| 63 | 31.948203821497902 | 2.06839714949771 |
### Chart
| Category | DW | Q N (mg) |
|---|---|---|
| 0 | 62.33103702989848 | 5.39726911471266 |
| 14 | 65.57099820769119 | 4.5495922433784 |
| 27 | 56.929351844126174 | 3.35073178355817 |
| 63 | 33.45077738132691 | 2.04181362436855 |
*
***
**
ns
Nitrogen quantity (mg)
Nitrogen quantity (mg)
***
**
Nitrogen content (%)
Nitrogen content (%)
A
AB
A
B
A
A
AB
B
DAF
DAF
Figure S2. Nitrogen content and quantity in pea leaves from the beginning of flowering until the end of the life cycle. Data are mean ± standard error (n=8 plants) of nitrogen content (%) and quantity (mg) in upper (A) and lower (B) leaves of plants supplied with nitrate (N+) or deprived of nitrate (N-). Different letters indicate significant differences (ANOVA and SNK test, P<0.05) in nitrogen quantity between two stages. Stars indicate significant differences in nitrogen content between two stages (ANOVA and SNK test, P>0.05, * P<0.05, ** P<0.01, *** P<0.001, ns: non-significant). DAF, days after flowering.

## Slide 3
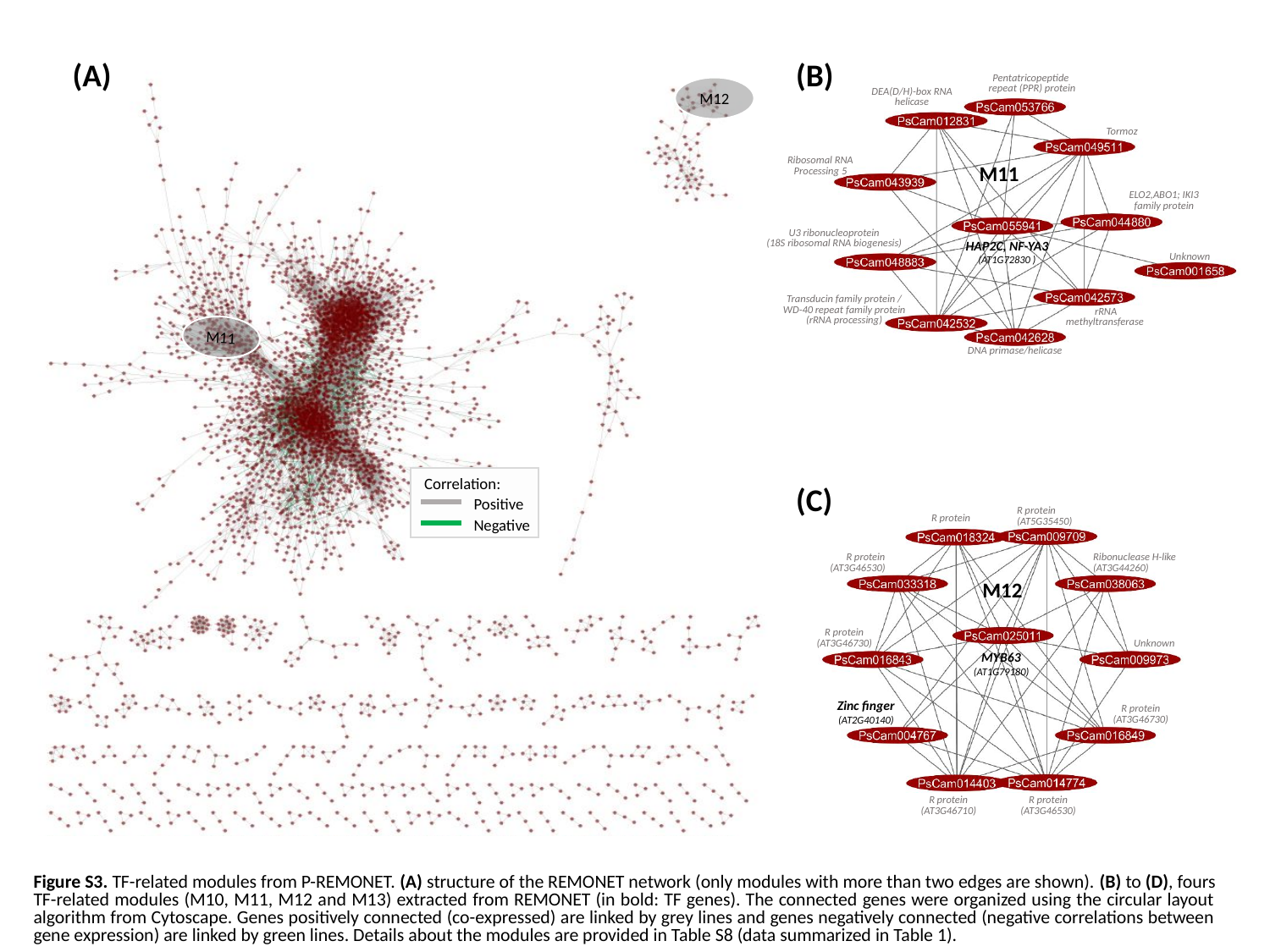

M12
M11
(A)
(B)
Pentatricopeptide
repeat (PPR) protein
DEA(D/H)-box RNA helicase
Tormoz
Ribosomal RNA Processing 5
M11
ELO2,ABO1; IKI3 family protein
U3 ribonucleoprotein
(18S ribosomal RNA biogenesis)
HAP2C, NF-YA3
(AT1G72830 )
Unknown
Transducin family protein /
WD-40 repeat family protein
(rRNA processing)
rRNA
methyltransferase
DNA primase/helicase
Correlation:
Positive
Negative
(C)
R protein
(AT5G35450)
R protein
R protein
(AT3G46530)
Ribonuclease H-like
(AT3G44260)
M12
R protein (AT3G46730)
Unknown
MYB63
(AT1G79180)
Zinc finger
(AT2G40140)
R protein (AT3G46730)
R protein
(AT3G46710)
R protein
(AT3G46530)
Figure S3. TF-related modules from P-REMONET. (A) structure of the REMONET network (only modules with more than two edges are shown). (B) to (D), fours TF-related modules (M10, M11, M12 and M13) extracted from REMONET (in bold: TF genes). The connected genes were organized using the circular layout algorithm from Cytoscape. Genes positively connected (co-expressed) are linked by grey lines and genes negatively connected (negative correlations between gene expression) are linked by green lines. Details about the modules are provided in Table S8 (data summarized in Table 1).

## Slide 4
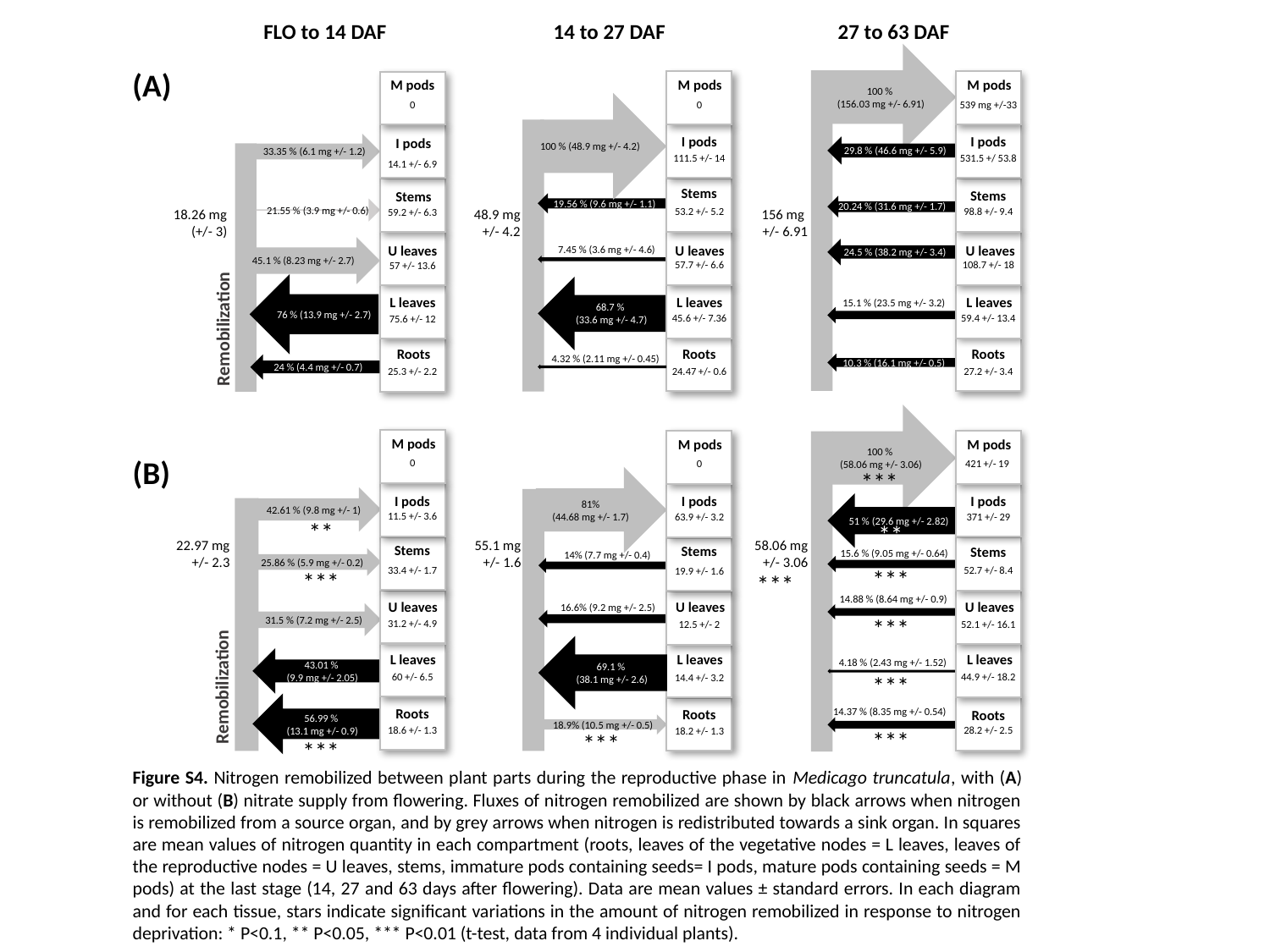

FLO to 14 DAF
14 to 27 DAF
27 to 63 DAF
100 %
(156.03 mg +/- 6.91)
(A)
M pods
M pods
M pods
539 mg +/-33
0
0
100 % (48.9 mg +/- 4.2)
531.5 +/ 53.8
111.5 +/- 14
14.1 +/- 6.9
I pods
I pods
I pods
33.35 % (6.1 mg +/- 1.2)
29.8 % (46.6 mg +/- 5.9)
Stems
53.2 +/- 5.2
98.8 +/- 9.4
59.2 +/- 6.3
Stems
Stems
20.24 % (31.6 mg +/- 1.7)
19.56 % (9.6 mg +/- 1.1)
21.55 % (3.9 mg +/- 0.6)
18.26 mg (+/- 3)
48.9 mg
+/- 4.2
156 mg
+/- 6.91
108.7 +/- 18
57.7 +/- 6.6
57 +/- 13.6
U leaves
U leaves
U leaves
7.45 % (3.6 mg +/- 4.6)
45.1 % (8.23 mg +/- 2.7)
24.5 % (38.2 mg +/- 3.4)
76 % (13.9 mg +/- 2.7)
68.7 %
(33.6 mg +/- 4.7)
59.4 +/- 13.4
45.6 +/- 7.36
75.6 +/- 12
L leaves
L leaves
L leaves
15.1 % (23.5 mg +/- 3.2)
Remobilization
27.2 +/- 3.4
24.47 +/- 0.6
25.3 +/- 2.2
Roots
Roots
Roots
4.32 % (2.11 mg +/- 0.45)
10.3 % (16.1 mg +/- 0.5)
24 % (4.4 mg +/- 0.7)
100 %
(58.06 mg +/- 3.06)
M pods
M pods
M pods
0
0
421 +/- 19
(B)
***
 81%
(44.68 mg +/- 1.7)
11.5 +/- 3.6
371 +/- 29
63.9 +/- 3.2
I pods
I pods
I pods
42.61 % (9.8 mg +/- 1)
51 % (29.6 mg +/- 2.82)
**
**
58.06 mg
+/- 3.06
22.97 mg
+/- 2.3
55.1 mg
+/- 1.6
Stems
Stems
Stems
33.4 +/- 1.7
52.7 +/- 8.4
19.9 +/- 1.6
15.6 % (9.05 mg +/- 0.64)
 14% (7.7 mg +/- 0.4)
25.86 % (5.9 mg +/- 0.2)
***
***
***
14.88 % (8.64 mg +/- 0.9)
31.2 +/- 4.9
52.1 +/- 16.1
12.5 +/- 2
U leaves
U leaves
U leaves
 16.6% (9.2 mg +/- 2.5)
31.5 % (7.2 mg +/- 2.5)
***
69.1 %
(38.1 mg +/- 2.6)
60 +/- 6.5
44.9 +/- 18.2
L leaves
L leaves
L leaves
14.4 +/- 3.2
43.01 %
(9.9 mg +/- 2.05)
4.18 % (2.43 mg +/- 1.52)
Remobilization
***
18.6 +/- 1.3
28.2 +/- 2.5
18.2 +/- 1.3
Roots
14.37 % (8.35 mg +/- 0.54)
Roots
Roots
56.99 %
(13.1 mg +/- 0.9)
 18.9% (10.5 mg +/- 0.5)
***
***
***
Figure S4. Nitrogen remobilized between plant parts during the reproductive phase in Medicago truncatula, with (A) or without (B) nitrate supply from flowering. Fluxes of nitrogen remobilized are shown by black arrows when nitrogen is remobilized from a source organ, and by grey arrows when nitrogen is redistributed towards a sink organ. In squares are mean values of nitrogen quantity in each compartment (roots, leaves of the vegetative nodes = L leaves, leaves of the reproductive nodes = U leaves, stems, immature pods containing seeds= I pods, mature pods containing seeds = M pods) at the last stage (14, 27 and 63 days after flowering). Data are mean values ± standard errors. In each diagram and for each tissue, stars indicate significant variations in the amount of nitrogen remobilized in response to nitrogen deprivation: * P<0.1, ** P<0.05, *** P<0.01 (t-test, data from 4 individual plants).
